# Growth and prevalence of feeding difficulties in children with Robin sequence: a retrospective cohort study

**DOI:** 10.1007/s00784-016-1996-8

**Published:** 2016-11-21

**Authors:** Emma C. Paes, Iris A.C. de Vries, Wouter M. Penris, Karlijn H. Hanny, Selma W. Lavrijsen, Elselien K. van Leerdam, Maaike M. Rademaker, Esther S. Veldhoen, Rene M.J.C. Eijkemans, Moshe Kon, Corstiaan C. Breugem

**Affiliations:** 1 Department of Pediatric Plastic Surgery, Wilhelmina Children’s Hospital, University Medical Centre, PO Box 85500, 3508 GA Utrecht, The Netherlands; 2 Faculty of Behavioral and Social Sciences, University of Groningen, Groningen, The Netherlands; 3 Department of Pediatrics, Wilhelmina Children’s Hospital, Utrecht, The Netherlands; 4 Department of Biostatistics and Research Support, Julius Centre, University Medical Centre, Utrecht, The Netherlands; 5 Department of Plastic Surgery, Meander Medical Center, Amersfoort, The Netherlands

**Keywords:** (Pierre) Robin sequence, Cleft palate, Treatment, Feeding difficulties, Growth, Weight, Systematic review

## Abstract

**Objectives:**

In addition to breathing problems, patients with Robin sequence (RS) often encounter feeding difficulties (FD). Data regarding the occurrence of FD and possible influencing factors are scarce. The study aim was to elucidate these factors to improve treatment strategies.

**Material and methods:**

A retrospective comparative cohort study was conducted, consisting of 69 infants diagnosed with both RS and a cleft palate and 64 isolated cleft palate only (iCPO) infants. Data regarding FD, growth, and airway intervention were collected during the first 2 years of life. A systematic review of the literature was conducted to identify reported FD in RS patients.

**Results:**

RS patients had more FD (91 %) than iCPO patients (72 %; *p* = 0.004). Also, nasogastric (NG)-tube feeding was necessary more frequently and for a longer period (both *p* < 0.001). Growth was lower in RS than iCPO infants (*p* = 0.008) and was not affected by the kind of airway management (conservative/surgical; *p* = 0.178), cleft palate grade (*p* = 0.308), or associated disorders (*p* = 0.785). By contrast, surgical intervention subtype did significantly affect growth. Mean reported FD for RS in the literature is 80 % (range = 47–100 %), and 55 % (range = 11–100 %) of infants need NG-tube feeding.

**Conclusions:**

FD is present in a large proportion of infants with RS, which indicates the need for early recognition and proper treatment to ensure optimal growth. Growth during the first 2 years of life is significantly lower in RS patients than iCPO patients, which indicates the need for careful attention and long-term follow-up.

**Clinical relevance:**

This study indicates the need for early recognition and proper treatment of FD in RS to ensure optimal growth. In addition, growth needs careful attention and long-term follow-up.

## Introduction

Although preceded by several earlier reports [1, 2], the French stomatologist Pierre Robin is credited as the first to draw attention to a symptom triad of breathing problems, glossoptosis, and micrognathia, known as Robin sequence (RS) [3, 4]. RS occurs in 1 in 8000 [5, 6] to 14,000 [7], depending on geography, ethnicity, and definition. Associated syndromes or anomalies coincide with RS in the majority of cases [8], and a concomitant cleft palate (CP) may exist but is not a required feature of RS [9–15].

Besides the varying degrees of respiratory problems, infants with RS frequently have feeding difficulties (FD) [16]. Swallowing difficulties directly related to the glossoptosis [16] and oroesophageal motor disorders caused by primary brainstem dysfunction [17] have been described as causes. FD is also a common feature in infants with a CP [18, 19]. These physiological abnormalities impede successful coordination of breathing, sucking, and swallowing. In infants with RS and a concomitant CP, these features can negatively affect the feeding process and there is a considerable risk of failure to thrive [20]. Consequently, these patients are often in need of nasogastric (NG)-tube feeding [21].

Although the majority of infants with RS and airway obstruction can be treated conservatively [22–26], surgical measures such as tongue lip adhesion (TLA) [27, 28], tracheotomy [29, 30], or mandibular distraction osteogenesis (MDO) [31–33] may be necessary. While the effect of these interventions on the obstructed airway has been frequently reported, information regarding the influence on FD is limited [16, 34].

To the best of our knowledge, this study is the first to identify factors that influence feeding and growth in RS and describe weight gain in the first 2 years of life. By obtaining a better understanding of all the facets of this condition, the treatment of these infants can be further optimized.

## Patients and methods

### Retrospective cohort study

#### Baseline characteristics

All infants diagnosed with RS (defined as the presence of micrognathia, glossoptosis, and signs of airway obstruction) and a concomitant CP treated at the Wilhelmina Children’s Hospital Utrecht, the Netherlands between 1996 and 2012 were included in the study group. All infants diagnosed with an isolated CP only (iCPO), without associated anomalies, were included in the control group. A retrospective analysis of the medical records during the first 2 years of life was conducted. Ethics committee approval was obtained to conduct this study (reference number WAG/th/14/020120).

The following variables were extracted from the medical files: gender, gestational age (GA), birth weight, grade of CP (grade 1–4) [35], and airway and nutritional treatment. In the study group, a subdivision was made between non-isolated RS infants (i.e., diagnosis of an additional syndrome, associated anomalies, or chromosomal defects) and isolated RS infants. Airway intervention was either conservative (i.e., prone/side positioning and possible use of supplemental oxygen, nasopharyngeal airway (NPA), oropharyngeal airway (mayotube), or continuous positive airway pressure) or surgical. The surgical intervention group was further divided into five subtypes: MDO, TLA, tracheotomy (Tr), TLA + Tr, and MDO + Tr.

#### Feeding and growth

FD were defined as (parentally) reported feeding problems, such as choking, regurgitation, gagging, distress, long-lasting feedings (≥30 min), impaired intake, and/or nasal regurgitation [36]. FD can lead to insufficient weight gain, failure to thrive, need for NG-tube feeding, and can potentiate airway or respiratory compromise [14]. Medical records and growth charts were thoroughly analyzed. In addition, parents received a phone call requesting participation in a short questionnaire about FD.

The following variables were collected: presence of FD, need and duration of NG-tube feeding, and weight at birth and at 1, 3, 6, 9, 11, 14, 17, and 24 months of age (if available). Growth was measured as a change between the consecutive measurements at these nine time points. In addition, normal weight standard deviation scores of healthy controls were collected [37]. In the surgical intervention subtypes, besides total NG-tube duration, the postoperative (i.e., after the airway intervention) NG-tube duration was also collected.

#### Statistical analysis

Data were analyzed using SPSS 20.0 (IBM SPSS, NY, USA). For interactions between nominal variables, chi-squared tests were used. To compare interactions between nominal and interval variables, *t* tests and one-way ANOVA were computed. For two interval variables, two-way Pearson correlations were calculated. To compare growth, linear mixed model analysis was performed to model the repeated measurements data. In non-normally distributed data, non-parametric tests were used: Mann-Whitney *U* and Kruskal-Wallis *H*.

### Systematic literature review

**Fig. 1:**
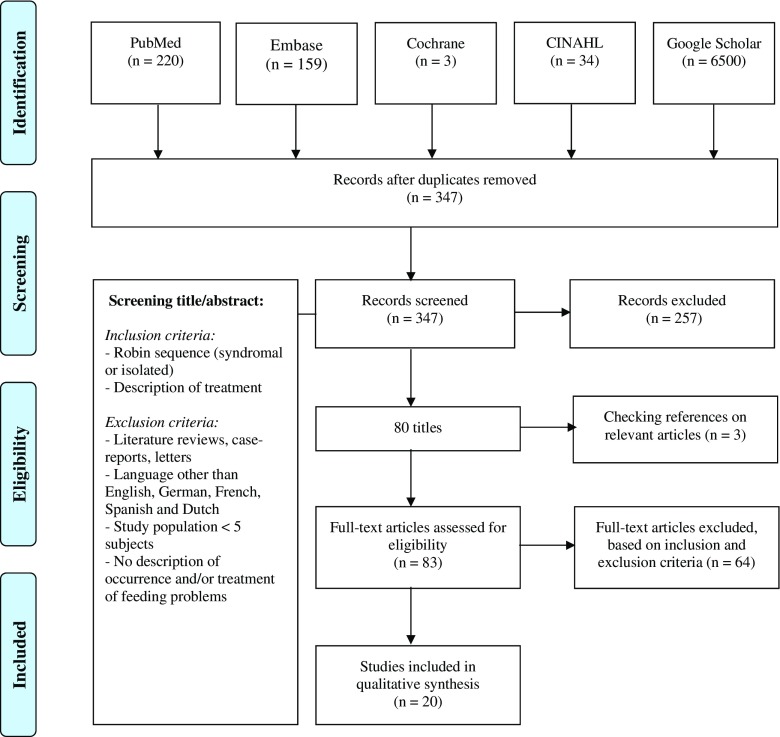
PRISMA flow diagram of the literature search

A systematic review of the literature was performed according to the Preferred Reporting Items for Systematic Reviews and Meta-Analysis (PRISMA) guidelines to assess current data on the combination of RS and FD [38]. Electronic databases were searched using specific keywords (Table 1) for articles published between July 1967 and August 2014, according to the search and inclusion processes as illustrated in Fig. 1. All relevant level I to level IV articles [39] were included for further analysis (Table 2).

**Table 1:** Search strategy of the systematic literature reviews in the databases used

Database	Search query
PubMed	(((“Pierre Robin Syndrome”[Mesh]) OR (pierre robin syndrome[tiab] OR pierre robin sequence[tiab] OR PRS[tiab] OR pierre robin[tiab] OR robin sequence*[tiab]))) AND (((“Feeding Behavior”[Mesh]) OR “Eating Disorders”[Mesh]) OR (feeding behavior*[tiab] OR feeding behaviour*[tiab] OR feed*[tiab] OR nutrition*[tiab] OR feeding difficult*[tiab] OR eating difficult*[tiab] OR feeding problem*[tiab] OR eating problem*[tiab] OR eating disorder*[tiab]))
Embase	(((‘pierre robin syndrome’:ab,ti OR ‘pierre robin sequence’:ab,ti OR ‘prs’:ab,ti OR ‘pierre robin syndromes’:ab,ti OR ‘pierre robin sequences’:ab,ti) OR ‘pierre robin syndrome’/exp) AND ((‘feeding behaviour’:ab,ti OR ‘feeding behaviours’:ab,ti OR ‘feeding behavior’:ab,ti OR ‘feed’:ab,ti OR ‘feeding’:ab,ti OR ‘nutrition’:ab,ti OR ‘nutritions’:ab,ti OR ‘feeding difficulty’:ab,ti OR ‘feeding difficulties’:ab,ti OR ‘feeding problem’:ab,ti OR ‘feeding problems’:ab,ti OR ‘eating problem’:ab,ti OR ‘eating problems’:ab,ti OR ‘eating difficulty’:ab,ti OR ‘eating difficulties’:ab,ti OR ‘eating disorder’:ab,ti OR ‘eating disorders’:ab,ti) OR ‘feeding behavior’/exp. OR ‘child nutrition’/exp. OR ‘nutritional disorder’/exp. OR ‘feeding disorder’/exp)) AND [embase]/lim NOT [medline]/lim
Cochrane library	Feeding behaviour* OR feeding behavior* OR feed* OR nutrition* OR feeding difficult* OR eating difficult* OR feeding problem* OR eating problem* OR eating disorder*:ti OR feeding behavior* OR feeding behavior* OR feed* OR nutrition* OR feeding difficult* OR eating difficult* OR feeding problem* OR eating problem* OR eating disorder*:ab AND pierre robin syndrome OR pierre robin sequence OR PRS OR pierre robin OR robin sequence*:ti OR pierre robin syndrome OR pierre robin sequence OR PRS OR pierre robin OR robin sequence*:ab
CINAHL	(TI pierre robin syndrome OR pierre robin sequence OR PRS OR pierre robin OR robin sequence* OR AB pierre robin syndrome OR pierre robin sequence OR PRS OR pierre robin OR robin sequence*) AND (S1 AND S2) TI (pierre robin syndrome OR pierre robin sequence OR PRS OR pierre robin OR robin sequence*) OR AB (pierre robin syndrome OR pierre robin sequence OR PRS OR pierre robin OR robin sequence*) TI (Feeding behaviour* OR feeding behavior* OR feed* OR nutrition* OR feeding difficult* OR eating difficult* OR feeding problem* OR eating problem* OR eating disorder) OR AB (Feeding behaviour* OR feeding behavior* OR feed* OR nutrition* OR feeding difficult* OR eating difficult* OR feeding problem* OR eating problem* OR eating disorder)
Google Scholar	pierre robin sequence OR pierre robin syndrome OR PRS AND feeding difficulties OR feeding problems OR nutrition

*CINAHL* Cumulative Index to Nursing and Allied Health Literature

**Table 2:** Comprehensive results of the systematic literature review

Article	Population	Reported FD	Treatment *n* (%)			Duration	Growth/weight gain	Intervention	Effect on FD
			NG-tube	Gastrostomy	Other				
Abadie et al. [40]	- *N* = 66 - Gl, RG, CP - iRS	98 %	34 (52 %)	–	–	3 months	Normal in 97 %	Tr	26 % (*n* = 5) bottle feeding after Tr
Anderson et al. [35]	- *N* = 12 - Gl, MG, CP, ORD - iRS, sRS	100 %	12 (100 %)	–	–	nm	Mean of 28 g/day	NPA	nm
Baujat et al. [17]	- *N* = 35 - Gl, RG, CP - iRS, sRS	100 %	30 (86 %)	–	–	8.6 months (±1.7 months)	nm	nm	nm
Butow et al. [24]	- *N* = 143 - Gl, MG/RG, CP - iRS, sRS	52 %	27 (19 %)	2 (6 %)	Suction and drinking plate (*n* = 134, 94 %)	nm	nm	Suction and drinking plate	26 % (*n* = 35) NG-tube feeding or gastrostomy needed
Cruz et al. [41]	- *N* = 43 - Gl, MG, CP - iRS, sRS	Nm	5 (11 %)	12 (26 %)	–	NG-tube ≥3 months	nm	Tr/TLA	nm
Daniel et al. [42]	- *N* = 39 - Gl, MG, ORD - iRS, sRS	82 %	32 (82 %)	–	–	nm	3230 ± 590 g (birth) 8890 ± 1290 g (12 months)	MDO, NPA, CPAP	nm
Evans et al. [43]	- *N* = 115 - Gl, RG/MG, CP, ORD - iRS, sRS	95 %	27 (23 %)	49 (43 %)	Nipple (*n* = 40, 35 %)	nm	nm	NPA, Tr, TLA	nm
Glynn et al. [30]	- *N* = 69 - Gl, MG, CP - iRS, sRS	70 %	48 (70 %)	2 (3 %)	–	3 months (3 weeks–6 months)	nm	NPA, Tr, TLA	nm
Gozu et al. [44]	- *N* = 20 - Gl, MG, CP - iRS, sRS	60 %	12 (60 %)	–	–	nm	nm	MDO	nm
Hamdi et al. [45]	- *N* = 30 - CP, ORD, FD - iRS, sRS	100 %	30 (100 %)	–	–	57 days (1–120 days)	nm	TLA, Tr, removable palatal appliance	nm
Li et al. [46]	- *N* = 82 - Gl, MG, CP - iRS, sRS	Nm	46 (42 %)	–	Obturator plate/CP bottles (*n* = 36, 44 %)	nm	nm	NPA ,TLA, Tr	nm
Lidsky et al. [47]	- *N* = 82 - Gl, MG, CP - iRS, sRS	Nm	–	17 (25 %)	–	nm	nm	Tr, MDO	Reduction of need for feeding intervention in iRS
Maas et al. [48]	- *N* = 151 - MG/RG and ORD/Gl/FD/snoring/hypoxemia/failure to thrive/syndrome or CP - iRS, sRS	89 %	76 (55 %); data only provided at time of discharge, after a mean of 19 (11–38) days	–	Feeding plate (*n* = 56, 37 %)	nm	Decrease of 0.59 (1.08–0.11) in SD score for weight, during admission	Orthodontic plate therapy, CPAP, Tr, TLA, mandibular traction, functional therapy (e.g., Castillo Morales)	Infants with pre-epiglottic baton plate demonstrated better weight gain during admission
Meyer et al. [49]	- *N* = 74 - Gl, MG, CP - iRS, sRS	50 %	19 (26 %)	18 (24 %)	–	nm	nm	Tr, MDO, NPA	nm
Smith et al. [50]	- *N* = 60 - Gl, MG/RG, CP and/or ORD - iRS, sRS	62 %	17 (28 %)	20 (33 %)	–	26 % 0–3 months, 31 % 4–18 months, 43 % >18 months	Successful oral diet in 89 % of patients after 3 years FU	Tr or MDO	nm
Stubenitsky et al. [51]	- *N* = 44 - Gl, MG/RG, CP, ORD - iRS	100 %	30 (68 %)	1 (2 %)	Reflux therapy (*n* = 27, 61 %)	nm	Mean weight gain 722 g in 4 weeks	NPA	nm
van den Elzen et al. [52]	- *N* = 74 - MG, CP, and/or Gl - iRS, sRS	68 %	30 (41 %)	2 (3 %)	Central venous line (*n* = 1, 1 %)	nm	Significant number of infants (24 %, *p* = 0.002) with body weight < P10 at age 6–24 months compared with healthy controls	Tr	nm
van Lieshout et al. [53]	- *N* = 59 - MG/RG and ORD - iRS, sRS	47 %	25 (34 %)	3 (4 %)	–	nm	nm	Tr, MDO	nm
Vatlach et al. [5]	- *N* = 82 - MG/RG with at least: ORD, Gl, FD, or CP - iRS, sRS	83 %	40 (49 %)	–	Haberman feeder (*n* = 16, 20 %); regular nipple (*n* = 21, 26 %)	nm	SDS of weight decreased from −0.72 at admission to −1.46 at discharge (*p* < 0.05)	Tr, MDO, CPAP, NPA, orthodontic plate therapy, TLA, functional therapy (e.g. Castillo Morales)	nm
Wagener et al. [54]	- *N* = 22 - MG, Gl, ORD, CP - iRS, sRS	100 %	22 (100 %)	–	–	4 months (1 week–11 months)	56 % ↑ weight; 44 % ↓ weight	NPA	nm

*FD* feeding difficulties*, NG-tube* nasogastric tube, *MG/RG* micrognathia/retrognathia, *Gl* glossoptosis, *ORD* obstructive respiratory distress, *CP* cleft palate, *iRS* isolated Robin sequence, *sRS* syndromic Robin sequence, *Tr* tracheotomy, *TLA* tongue lip adhesion, *MDO* mandibular distraction osteogenesis, *NPA* nasopharyngeal airway, *CPAP* continuous positive airway pressure, *nm* not mentioned

## Results

### Baseline characteristics

Sixty-nine RS patients (study group) and 64 consecutive iCPO patients (control group) were included. The study group included significantly more patients with a grade 3/4 CP than the control group (*p* < 0.001). The majority of the study group (54 %; *n* = 37) was made up of non-isolated RS patients. Of these, more than half had an associated syndrome (51 %, *n* = 19), Stickler syndrome (*n* = 9) being the most common (Table 3).

**Table 3:** Baseline characteristics of the patients in the study and control groups treated in the Wilhelmina Children’s Hospital between 1996 and 2012

Variable		Study group (RS) *n* (%)	Control group (iCPO) *n* (%)	*p* value*
Total number of patients		69	64	
Sex	Male	32 (46 %)	23 (36 %)	0.22
	Female	37 (54 %)	41 (64 %)	
Gestational age	<37 weeks	8 (12 %)	7 (11 %)	0.91
	≥37 weeks	61 (88 %)	57 (89 %)	
Birth weight (g)		Mean = 3217 SD = 669	Mean = 3302 SD = 556	0.44
Grade of CP^a^	1. Submucous cleft or bifid uvula	3 (4 %)	9 (14 %)	<0.001
	2. Soft palate only	10 (15 %)	27 (42 %)	
	3. Soft palate and segment of hard palate	38 (56 %)	18 (28 %)	
	4. Total palate up to incisive foramen	17 (25 %)	10 (16 %)	
Associated disorders	Isolated RS 32 (46 %) Non-isolated RS 37 (54 %)			0.06
	Stickler syndrome	9		
	Treacher Collins syndrome	2		
	Spondyloepiphyseal dysplasia	1		
	4q deletion syndrome	1		
	Van der Woude syndrome	1		
	Osteopathia striata with cranial sclerosis	1		
	Goldberg–Shprintzen syndrome	1		
	Yunis–Varon syndrome	1		
	Auriculo-condylar syndrome	1		
	Hemifacial microsomia	1		
	Other	18		

*RS* Robin sequence, *iCPO* isolated cleft palate only, *SD* standard deviation, *CP* cleft palate

*p value < 0.05 was considered statistically significant

^a^Modified from Jensen et al. cleft palate classification (1988) [34], according to the division made in the Dutch Cleft Registry database

### FD and NG-tube feeding

RS patients expressed FD (91 %; *n* = 63) more than iCPO patients (72 %; *n* = 38, *p* = 0.004). In RS and iCPO patients with FD, a highly significant association was found in CP grade between the two groups (*p* < 0.001); while a grade 3 and 4 CP was most common in RS patients with FD (grade 3 = 60 %, *n* = 37; grade 4 = 23 %, *n* = 14), a grade 2 CP was most common in iCPO patients with FD (50 %; *n* = 19; Table 4). In a logistic regression analysis controlled for CP grade, presence of FD was still significantly associated with the RS patient group (*p* = 0.005).

**Table 4:** Association of feeding difficulties and NG-tube feeding between the study and the control groups

Variable		Study group (RS)	Control group (iCPO)	*p* value*
Total number of patients		69	64	
*With* *feeding difficulties*		63 (91 %)	38 (72 %)	0.004
Sex	Male	27 (43 %)	15 (40 %)	0.74
	Female	36 (57 %)	23 (61 %)	
Gestational age	<37 weeks	7 (11 %)	5 (13 %)	0.76
	≥37 weeks	56 (89 %)	33 (87 %)	
Birth weight (g)		Mean = 3237 SD = 644	Mean = 3289 SD = 586	0.68
Grade of CP	1. Submucous cleft or bifid uvula	2 (3 %)	3 (8 %)	<0.001
	2. Soft palate only	9 (15 %)	19 (50 %)	
	3. Soft palate and segment of hard palate	37 (60 %)	10 (26 %)	
	4. Total palate up to incisive foramen	14 (23 %)	6 (16 %)	
Associated disorders	Non-isolated RS^b^	36 (57 %)	NA	NA
	Isolated RS	27 (43 %)		
*With NG-tube feeding*		55 (80 %)	12 (19 %)	<0.001
NG-tube feeding duration (days)		Median = 59.00 Mean rank = 33.13	Median = 9.56 Mean rank = 9.72	<0.001
Sex	Male	24 (44 %)	7 (58 %)	0.36
	Female	31 (56 %)	5 (42 %)	
Gestational age	<37 weeks	7 (13 %)	4 (33 %)	0.08
	≥37 weeks	48 (87 %)	8 (67 %)	
Birth weight (g)		Mean = 3217 SD = 661	Mean = 3039 SD = 733	0.41
Grade of CP^a^	1. Submucous cleft or bifid uvula	1 (2 %)	0 (0 %)	0.23
	2. Soft palate only	9 (17 %)	5 (42 %)	
	3. Soft palate and segment of hard palate	32 (59 %)	4 (33 %)	
	4. Total palate up to incisive foramen	12 (22 %)	3 (25 %)	
Associated disorders	Non-isolated RS^b^	32 (58 %)	NA	NA
	Isolated RS	16 (42 %)		

Due to missing values, the results for certain variables presented in this table do not correspond with the total participants per investigated variable

*RS* Robin sequence, *iCPO* isolated cleft palate only, *SD* standard deviation, *NG-tube* nasogastric tube, *NA* not applicable

*p value < 0.05 was considered statistically significant

^a^Modified from Jensen et al. cleft palate classification (1988) [34], according to the division made in the Dutch Cleft Registry database

^b^Presence of a syndrome or other associated anomalies or chromosomal defects

NG-tube feeding was more often necessary in RS patients (80 %; *n* = 55) than iCPO patients (19 %; *n* = 12, *p* < 0.001). Furthermore, NG-tube feeding lasted longer in RS patients (median 59.0 days in study group vs. median 9.6 days in control group, *p* < 0.001). There was no significant association between the grade of CP (1–4) and the incidence of NG-tube feeding (*p* = 0.23; Table 4). NG-tube duration of the isolated (125 days; SD 203) and non-isolated (125 days; SD 171) RS patients did not differ significantly (*p* = 0.996).

### Growth

**Fig. 2:**
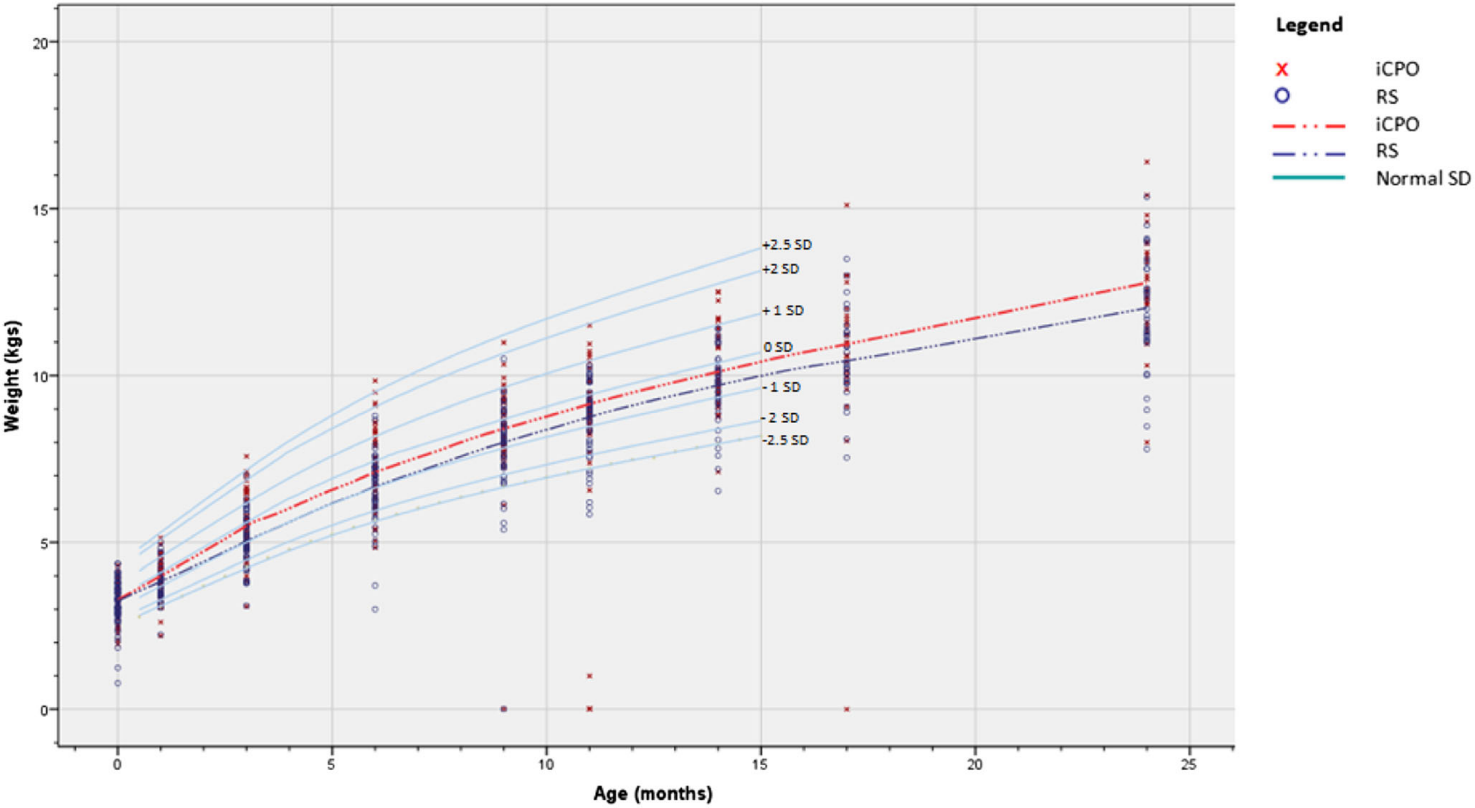
Growth in the first 2 years of life in the study and control group, compared with normal SD values of healthy Dutch infants [37]

Birth weights of the two groups were comparable (iCPO group 3302 g vs. RS group 3217 g, *p* = 0.41). However, the iCPO group showed a significantly higher overall growth over the time points 1–9 (birth to 24 months of age) than the RS group (*p =* 0.008). This increased growth in the iCPO group was also visible when separately analyzing time points 1–4 (birth to 6 months of age) and 5–9 (9–24 months of age; Table 5 and Fig. 2). When additionally controlling for grade of CP over time points 1–9, this difference remained significant (*p* = 0.030).

**Table 5:** Variable effects on growth measured by weight (in grams) over the nine measured time points

Variable effects on growth^a^		EMM (g)	SE	95 % CI (upper bound–lower bound)	*p* value*
Time points 1–9 (birth to 24 months of age)	iCPO	5620	96	5263–5678	0.008
	RS	5261	95	5240–5581	
Time points 1–4 (birth to 6 months of age)	iCPO	3805	65	3676–3934	0.044
	RS	3619	65	3471–3746	
Time points 5–9 (9 to 24 months of age	iCPO	9833	143	9551–10,114	0.026
	RS	9390	138	9119–9661	
Time points 1–9^b^ (birth to 24 months of age)	iCPO	5588	106	5380–5796	0.030
	RS	5268	112	5047–5490	5268
Feeding difficulties^c, f^	Yes	6902	87	6147–6488	0.467
	No	6767	192	6094–6849	
NG-tube feeding^c, f^	Yes	6584	189	−396–346	0.893
	No	6559	189	−346–396	
Grade of CP^c, f^	1. Submucous cleft or bifid uvula	5468	237	5019–5953	0.308
	2. Soft palate only	5540	130	5283–5797	
	3. Soft palate and segment of hard palate	5489	107	5278–5699	
	4. Total palate up to incisive foramen	5198	146	4911–5486	
Associated disorders^d, f^	Isolated RS	6479	151	6181–6777	0.517
	Non-isolated RS^e^	6621	154	6317–6825	
Intervention type^d, f^	Surgical	6902	203	6504–7301	0.052
	Conservative	6484	179	6132–6836	
Surgical intervention subtype^d, f^	MDO	7965	188	7587–8344	0.007
	TLA	7720	336	7049–8391	
	Tr	8765	223	8317–9213	
	TLA + Tr	6423	752	4920–7927	
	MDO + Tr	8383	412	7555–9210	

Time points: weight at birth, 1, 3, 6, 9, 11, 14, 17, and 24 months of age

*EMM* estimated marginal means, *SE* standard error, *CI* confidence interval, *MDO* mandibular distraction osteogenesis, *TLA* tongue lip adhesion, *Tr* tracheotomy, *NG-tube* nasogastric tube, *RS* Robin sequence, *iCPO* isolated cleft palate only, *CP* cleft palate

*p value < 0.05 was considered statistically significant

^a^All measurements were controlled for gender

^b^Also controlled for grade of CP

^c^Also controlled for group

^d^Only analyzed within the RS group

^e^Presence of a syndrome or other associated anomalies or chromosomal defects

^f^For time points 1–9 (birth to 24 months of age)

Taken into consideration all nine time points, in the following analysis, both gender and group (iCPO vs. RS) were controlled for. Neither presence of FD nor the need for NG-tube feeding revealed significant effects on growth (*p =* 0.893 and *p* = 0.467, respectively). Furthermore, the grade of CP (1–4) did not significantly affect growth (*p* = 0.308; Table 5). Since a clinical interaction between the CP grade and group type could exist, this was also separately tested, showing that the interaction between the grade of CP (1–4) and group did not significantly affect growth (*p* = 0.112).

Within the RS group, neither the presence of associated disorders (isolated/non-isolated) nor intervention type (surgical/conservative) had a significant effect on growth (*p =* 0.517 and *p* = 0.052, respectively; Table 5).

### Interventions in the study group

While 40 (60 %) RS patients could be treated with conservative measures, in 27 infants (40 %), a surgical intervention was performed because of severe respiratory distress. MDO was pursued at a mean age of 36 days (SD 32) in 14 (52 %) of these cases, TLA (mean age = 77 days, SD 49) in 3 cases (11 %), and a tracheotomy was performed (mean age = 45, SD 27) in 7 cases (26 %). Finally, in one case after TLA, a tracheotomy was performed due to an unstable airway. In two other cases, MDO was performed after tracheotomy (Table 6). Background information on the decisional process can be found in earlier work [55].

**Table 6:** ᅟSignificant differences between all group interactions

		Conservative	Surgical	*p* value*	Surgical intervention subtype					
					MDO	TLA	Tr	TLA + Tr	MDO + Tr	*p* value*
Total number of patients		40 (60 %)	27 (40 %)		14 (52 %)	3 (11 %)	7 (26 %)	1 (4 %)	2 (7 %)	
Feeding difficulties, yes		34 (85 %)	27 (100 %)	0.04	14 (100 %)	3 (100 %)	7 (100.0 %)	1 (100.0 %)	2 (100 %)	n.a.
NG-tube feeding, yes		28 (63 %)	25 (93 %)	0.03	14 (100 %)	3 (100 %)	6 (86 %)	0 (0 %)	2 (100 %)	0.006
NG-tube duration (days)^a^		Median = 21.0	Mean = 72.0	0.011	Median = 36.5	Median = 183.0	Median = 461.5	NC	Median = 38.0	0.003
		Mean rank = 19.2	Mean rank = 29.4		Mean rank = 8.9	Mean rank = 18.3	Mean rank = 21.2	NC	Mean rank = 9.0	
Sex	Male	18 (45 %)	12 (44 %)	0.96	6 (43 %)	1 (33 %)	3 (43 %)	0 (0 %)	2 (100 %)	0.48
	Female	22 (55 %)	15 (56 %)		8 (59 %)	2 (67 %)	4 (57 %)	1 (100 %)	0 (0 %)	
Gestational age	<37 weeks	5 (13 %)	2 (7 %)	0.50	0 (0 %)	0 (0 %)	1 (14 %)	1 (100 %)	0 (0 %)	0.006
	≥37 weeks	35 (88 %)	25 (93 %)		14 (100 %)	3 (100 %)	6 (86 %)	0 (0 %)	2 (100 %)	
Birth weight (g)		Mean = 3139	Mean = 3409	0.10	Median = 3205	Median = 3785	Median = 3920	Median = 2680	Median = 3287	0.19
		SD = 713	SD = 521		Mean rank = 12.11	Mean rank = 16.33	Mean rank = 19.07	Mean rank = 2.5	Mean rank = 11.75	
Grade of CP^b^	1. Submucous cleft or bifid uvula	3 (8 %)	0 (0 %)	0.45	0 (0 %)	0 (0 %)	0 (0 %)	0 (0 %)	0 (0 %)	0.13
	2. Soft palate only	5 (13 %)	5 (19 %)		4 (31 %)	1 (33 %)	0 (0 %)	0 (0 %)	0 (0 %)	
	3. Soft palate and segment of hard palate	21 (53 %)	15 (58 %)		5 (39 %)	2 (67 %)	7 (100 %)	0 (0 %)	1 (50 %)	
	4. Total palate up to incisive foramen	11 (28 %)	6 (23 %)		4 (31 %)	0 (0 %)	0 (0 %)	1 (100 %)	1 (50 %)	
Associated disorders	Isolated	21 (53 %)	11 (41 %)	0.35	4 (29 %)	0 (0 %)	4 (57 %)	1 (100 %)	2 (100 %)	0.089
	Non-isolated^c^	19 (48 %)	16 (59 %)		10 (71 %)	3 (100 %)	3 (43 %)	0 (0 %)	0 (0 %)	

*MDO* mandibular distraction osteogenesis, *TLA* tongue lip adhesion, *Tr* tracheotomy, *NG-tube* nasogastric tube, *SD* standard deviation, *n.a.* not applicable, *NC* no calculation possible because of too few cases

*p value < 0.05 was considered statistically significant

^a^In the surgical intervention subtypes only the postoperative NG-tube duration was included

^b^Modified from Jensen et al. cleft palate classification (1988) [34], according to the division made in the Dutch Cleft Registry database

^c^Presence of a syndrome or other associated anomalies or chromosomal defects

FD showed a significant association with intervention (surgical/conservative; *p =* 0.04); while all surgically treated patients had FD (100 %), in the conservatively treated group 85 % expressed FD. Since 100 % of the surgically treated patients showed FD, further investigation of presence of FD within the type of surgical treatment was not possible (Table 6).

Surgically treated RS patients were significantly more often in need of NG-tube feeding than conservatively treated patients (93 vs. 63 %, *p* = 0.03). NG-tube feeding lasted significantly longer in surgically treated RS patients (median = 72.0 days; mean rank 29.4; *p* = 0.011) compared to conservatively treated patients (median = 21.0 days; mean rank 19.2). Surgical intervention subtype had a significant effect on postoperative duration of NG-tube feeding (*p* = 0.003), with a median of 36.5 days for MDO, 183.0 days for TLA, 461.5 days for Tr, and 38.0 days for MDO + Tr. A post hoc test revealed significant differences between all these group interactions (*p* < 0.05), except for TLA vs. Tr (*p* = 0.302), TLA vs. MDO + Tr (*p* = 0.083), and MDO vs. MDO + Tr (*p* = 0.874; Table 6).

The subtype of surgical intervention (MDO, TLA, Tr, TLA + Tr, or MDO + Tr) also demonstrated a significant effect on growth from birth to 24 months of age (*p* = 0.007); a post hoc test showed significant differences between MDO vs. Tr (*p* = 0.008), TLA vs. Tr (*p* = 0.012), Tr vs. TLA + Tr (*p* = 0.004), TLA + Tr vs. MDO (*p* = 0.05), and TLA + Tr vs. MDO + Tr (*p* = 0.029; Table 5).

### Systematic literature review

The literature search resulted in 347 unique titles. After initial screening, the full text of 80 potentially relevant articles was retrieved. References of these were checked, which provided three additional papers. These 83 texts were then analyzed by selection criteria and validity, yielding 20 articles (Table 2) [5, 17, 24, 30, 35, 40–54].

The selected reports included a mean of 65 patients (range = 22 [54]–151 [48]) with mixed isolated and non-isolated RS cases. In the majority, RS was defined as infants expressing micrognathia, glossoptosis, and a CP, while in the others, obstructive respiratory distress or FD were (optionally) included in the features of RS. An average of 80 % of cases expressed FD (range = 47 [53]–100 [17, 35, 45, 51] %). On average, NG-tube feeding was given to 55 % of the infants (range = 11 [41]–100 [35, 45, 54] %), and a gastrostomy in 17 % (range = 2 [24]–43 [43] %). Other feeding interventions described were special oral plates [24, 46, 48] or functional therapy (such as Castillo Morales) [5]. When mentioned, mean duration of NG-tube feeding varied between several weeks [30, 54] and 18 months [50].

## Discussion

### Feeding difficulties and growth

FD are an important and common symptom in RS, possibly leading to failure to thrive and developmental problems, if not recognized and treated in time [40]. Up to 73 % of infants with a CP have been reported to suffer FD [56]. The current study demonstrated that more FD were seen in infants with RS (91 %) than iCPO (72 %), also after controlling for CP grade (*p* = 0.005). All RS patients demonstrated a significantly lower growth than iCPO patients during the first 2 years of life irrespective of the treatment regime (*p* = 0.030) yet remained within the 0 SD (P50) and −1 SD (P16) line (Fig. 2). This finding is in line with other studies that have also demonstrated a lower birth weight in RS patients, compared to healthy individuals and iCPO patients [48, 52, 57, 58]. A hypothesis for the lower growth in RS infants is the presence of morphological characteristics as primary predisposing factors, which is supported by the finding that infants with CP have a tendency towards smaller cranial circumference [55]. Also genetic factors are of interest, especially the role of growth factors that might influence growth retardation in RS [57]. In addition, airway infections during 0–3 months of age negatively affect growth [59]. Finally, arguments for other origins of feeding disorders and subsequent growth retardation in patients with RS exist, such as primary brainstem dysfunction, or neuromotor disabilities, which might be more prevalent in RS than iCPO patients [17, 40]. Although CP patients have a lower weight than healthy controls [57], they tend to “catch-up” later in childhood [57, 59]. To date, no studies exist that describe growth patterns in patients with RS during a longer period. There is sparse evidence that severe functional feeding and respiratory disorders do not affect long-term developmental outcomes in infants with isolated RS of Stickler [21]. Still, longer follow-up studies of both isolated and non-isolated RS infant are needed to evaluate the cause, a possible catch-up in growth, and the effect of the lower weight on further (cognitive) development.

### Airway interventions

Growth was not affected by the type of airway intervention (conservative vs. surgical, *p =* 0.178); therefore, we hypothesize that adequate relief of airway obstruction is important to maintain adequate growth [60]. This finding is substantiated by similar findings of Daniel et al. [42], in which the degree of adequately treated OSA did not influence growth infants with RS. In the majority (58 %) of RS cases of our cohort, airway problems could be managed conservatively. Surgical options were only considered after NPA treatment failed [61]. Until 2006, either TLA or tracheotomy was performed. MDO has become our surgical procedure of preference in a supraglottic airway obstruction since 2006 [31, 61]. NG-tube feeding duration was significantly reduced after MDO treatment compared to the other surgical interventions, which corresponds with the results of others [34]. Lidksy and co-authors [47] show that also timing of surgery (i.e., MDO within 3 months) dramatically reduces the need for feeding interventions in isolated RS patients. Moreover, disappearance of gastroesophageal reflux has been demonstrated after MDO [16]. The positive effect of MDO on feeding capacity and growth has also been confirmed by others [44, 47, 62–64]. Interestingly, the RS infants of our cohort that received a tracheotomy had a significantly higher weight than the MDO or TLA group. This growth difference might result from a disproportionate presence of comorbidities or syndromes in the various surgical subtype groups, differing ages at surgery, or longer NG-tube durations in infants treated with tracheotomy. Also the radical resolution of their obstruction might add to a longer stay under medical control and nutritional support. Data of a recent German review demonstrates that improvement of weight is also possible by the appliance of a pre-epiglottic baton plate (PEBP) in RS infants with severe upper airway obstruction [48, 65]. Treatment with this orthodontic appliance was associated with a higher increase of weight than infants treated with prone positioning or tracheotomy [48]. Moreover, at discharge, a decrease in the proportion of infants requiring NG-tube feeding from 66 to 8 % was seen after PEBP treatment [65]. Although we have no experience with this conservative treatment method, the results of others are very promising and could be considered in an institutional algorithm.

### Associated anomalies

Infants with syndromes, such as in non-isolated RS, often express FD [66]. In the current study, a higher, although nonsignificant, presence of FD and NG-tube feeding frequency was seen in the non-isolated RS patients, compared with the isolated RS group. No significant effect of the presence of an associated disorder or syndrome was illustrated on growth, a finding that is in agreement with other studies [24, 42, 52, 67, 68]. In addition, the duration of NG-tube feeding in isolated vs. syndromic RS patients was not different. However, other underlying problems (e.g., neuromotor dysfunction) might persist longer, having a negative effect on feeding capacity despite adequate relief of airway problems [17, 47, 52]. In some studies, a higher rate of gastrostomy placement has been found in syndromic than isolated RS patients [41, 69]. In conclusion, we suggest that infants with a syndromic diagnosis need closer follow-up to monitor growth and feeding capacity [70].

### Analysis and treatment of FD

It is important to distinguish between respiratory-related FD and neuromotor disabilities that affect sucking and swallowing coordination [42, 71, 72]. Although numerous papers mention FD, there is still no scientific agreement about what they exactly encompass [36]. Consequently, these difficulties are regularly manifested in objectified measures, such as weight or the incidence of NG-tube feeding [36]. Also, several symptoms are proposed to confirm the presence of FD, such as dysphagia or gastroesophageal reflux (GER) [17, 40, 73–75]. There is no consensus about which investigations should be performed, and their validity is sometimes questionable [75]. We strongly believe it is important to define feeding issues together with parents, feeding therapists, and pediatricians as early as possible*.* NG-tube feeding should be started when there is insufficient weight gain [14]. In our institution, infants with FD were more likely to receive NG-tube feeding. When NG-tube feeding was adequately started, no differences in growth were seen between infants with reported FD and those without FD. The high frequency of FD in the iCPO group (72 %) contrasting with the low frequency of tube feeding in this group (12 %) is surprising. An explanation might be the parents’ interpretation of the presence of FD, which does not correspond to the criteria of pediatricians to start with NG-tube feeding. This finding might emphasize that the parental concerns need to be addressed and discussed by the medical team during the treatment of an infant with an iCPO. If GER is clinically suspected, a trial of reflux therapy is started, as the incidence of GER is known to be higher in RS [30]. Marquis et al. [76] stress the importance of hypercaloric feeding and demonstrate a quicker improvement in weight gain and relief of respiratory problems, compared with controls. In addition, many authors advise feeding-facilitating techniques, by stimulating the orofacial and tongue musculature and encouraging sucking to improve neuromuscular coordination by introducing small amounts of bottle feeding [5, 30, 54, 77]. Monitoring of urinary sodium has been suggested, as oral sodium supplementation in cases with a low urine sodium significantly improved weight gain in infants with RS [78]. Besides growth, maternal bonding [79], psychological well-being [80], and social and cognitive development [18, 19, 81] can be negatively influenced by FD and need to be monitored during follow-up.

### Strengths and limitations

The first limitation is the study’s retrospective nature. We did not examine nutritional status by using other anthropometric measurements, such as mid upper arm circumference and skin fold thickness [82, 83]. FD remains difficult to define and in addition to objective information retrieved from medical charts and growth charts we also included subjective information from parents. Consequently, differences in presence and severity of FD amongst the included infants existed and might also have been influenced by recall-bias. Other forms of promising therapy not used in our institution, such as Castillo Morales [5] or palatal plate therapy [45, 65, 84–87], have been described. Moreover, in our clinic, it is uncommon to perform a gastrostomy in children under 1 year of age; hence, we only provide data on usage of NG-tube. Finally, RS is a heterogenic disorder; thus, the distribution of syndromes or associated anomalies might influence the results. Strengths include that this is the first comparative study to report in detail on feeding issues and growth in two large cohorts over a 2-year study period, using weight at nine measuring moments as objective parameters and analyzing the influence of various parameters.

## Conclusion

In this retrospective study, the prevalence of FD was significantly higher and NG-tube feeding was more frequent and for a longer period in infants with RS than iCPO. Growth in the first 2 years of life was significantly lower in RS than iCPO infants, although following a steady curve between the 0 and −1 SD line compared with healthy counterparts. Neither presence of associated syndromes nor the type of intervention negatively affected growth, which might be explained by early recognition and treatment of FD in our cohort. The subtypes of surgical intervention did reveal a significant effect on growth, which might be caused by the heterogeneity of the treated infants. The cause of the lower growth in RS infants and the long-term effects, despite an apparent good treatment regime in terms of airway relief and monitoring of the intake, mandates further investigation. By gaining insight about this challenging patient group, treatment strategies can be optimized and expectations of caretakers and parents better managed.
